# Maine

**Published:** 1897-03

**Authors:** 


*Maine.* The Maine Cattle Commission formerly maintained that tuberculosis did not exist in that State. Recent experience has demonstrated that the disease is present in some herds. It is probable, however, that it is far less prevalent in this State than in southern New England, and it will be a much easier matter to deal with it. The Cattle Commission has for years endeavored to keep diseased cattle out of the State, and with much success. The whole matter will receive attention in the Legislature.—*New England Homestead.*

